# Autism spectrum disorder and attention-deficit/hyperactivity disorder in children with cerebral palsy: results from screening in a population-based group

**DOI:** 10.1007/s00787-020-01471-1

**Published:** 2020-01-11

**Authors:** Magnus Påhlman, Christopher Gillberg, Elisabet Wentz, Kate Himmelmann

**Affiliations:** 1 Gillberg Neuropsychiatry Centre, Institute of Neuroscience and Physiology, Sahlgrenska Academy, University of Gothenburg, Gothenburg, Sweden; 2 Regional Rehabilitation Centre, Queen Silvia Children’s Hospital, Gothenburg, Sweden; 3 Department of Psychiatry and Neurochemistry, Institute of Neuroscience and Physiology, Sahlgrenska Academy, University of Gothenburg, Gothenburg, Sweden; 4 Department of Pediatrics, Institute of Clinical Sciences, Sahlgrenska Academy, University of Gothenburg, Gothenburg, Sweden

**Keywords:** Cerebral palsy, Autism, ADHD, Children, Screening

## Abstract

Autism spectrum disorder (ASD) and attention-deficit/hyperactivity disorder (ADHD) are more common in children with cerebral palsy (CP) than in the general population, but may still be underdiagnosed. This study aimed to estimate screen-positive ASD and ADHD in a population-based group of 264 school-aged children with CP born 1999–2006 from the CP register of western Sweden. Validated parent-completed questionnaires were used at a median age of 12 years 11 months (range 8–17 years). Three different scales were used to detect signs of ASD and ADHD, respectively. Response rate was 88% (232/264). In 19 children, all in the most disabled group, the screening procedure was not feasible due to too few questionnaire items completed, leaving 213 for analyses. One third (74/213) of the children screened positive for ASD and half of the children (106/213) for ADHD, which was about twice as often as ASD/ADHD diagnoses had been clinically identified. Children with intellectual disability, epilepsy and/or impaired speech ability more often screened positive for ASD as well as ADHD. Severe motor impairment was more frequently associated with screen-positive ASD, but not ADHD. Neither sex nor CP type was associated with screen-positive ASD/ADHD. In conclusion, school-aged children with CP very often screened positive for ASD and/or ADHD. The prevalence of ASD and ADHD is most likely underestimated in children with CP. These screening findings require further investigations.

## Introduction

Cerebral palsy (CP) is the most common cause of motor disability in childhood, affecting about 2 per 1000 live births [1, 2]. CP is an umbrella term of conditions, heterogeneous in causation and manifestations, of non-progressive disturbances affecting the immature brain. Children with CP often have other functional disabilities and activity restrictions than the motor disability. In the most recent definition [3] problems relating to cognition, communication, behaviour, and sensation are mentioned. Several studies have described how the accompanying disabilities can be more disabling than the motor disability per se [4–7].

There is growing evidence that neuropsychiatric disorders, primarily autism spectrum disorder (ASD) and attention-deficit/hyperactivity disorder (ADHD), are more prevalent in children with CP than in the general population [8–16]. A recent systematic review does confirm this, but there are few population-based studies of children with CP [17].

A previous study by our group, where medical records of children in a population-based group with CP were scrutinised regarding identified neuropsychiatric disorders, supported these findings. ASD was diagnosed in 18% and ADHD in 21% [18]. These record-based numbers are most likely underestimations, and registered diagnosis rates depend on the documentation of neuropsychiatric diagnoses or descriptions of certain behaviours [15]. We also found a pattern of ASD and ADHD registered rates decreasing with increasing motor impairment, in contrast to the distribution of other impairments like intellectual disability (ID) and epilepsy, also suggesting underestimation of ASD and ADHD in more severe CP.

ASD screening studies in CP are scarce, often small and not population-based [9, 11]. A Norwegian screening study on psychiatric disorders in 7-year-old children with CP, showed 42% screened positive for ADHD [14]. To our knowledge, there has previously not been any population-based study that has actively assessed children with CP focusing on ASD and ADHD. The need for screening for ASD and ADHD with adequate tools is also emphasised by Craig et al. [17].

The first aim of this study was to estimate the prevalence of ASD and ADHD screen positivity in a well-defined population-based group of children with CP from the CP register of western Sweden, and compare with already identified diagnoses of ASD and ADHD. The second aim was to describe if and how screening positive for ASD and ADHD was associated with sex, gestational age, CP type, gross motor function, intellectual level, epilepsy and speech ability.

## Methods

### Participants

The study population, derived from the CP register of western Sweden, comprised eight birth-year cohorts of children and adolescents with CP born between 1999 and 2006 [1, 2], in the county of Västra Götaland, a region with 1.6 million inhabitants. This population-based group consisted of 264 children, 141 boys and 123 girls.

CP types were classified according to the Surveillance of Cerebral Palsy in Europe (SCPE); into unilateral spastic CP (USCP), bilateral spastic CP (BSCP), dyskinetic CP and ataxic CP [19]. Gross motor function was classified with the Gross Motor Function Classification System (GMFCS) [20].

Gestational-age groups were considered: extremely preterm (birth occurring at less than 28 completed gestational weeks), very preterm (28–31 weeks), moderately preterm (32–36 weeks) and term (37 completed weeks or more).

Intellectual level was defined as normal if IQ was ≥ 85, and borderline intellectual functioning if IQ was 70–84. Intellectual disability (ID, term according to Diagnostic and Statistical Manual of Mental Disorders (DSM)-5) [21] was defined according to International Classification of Diseases and Related Health Problems—Tenth Revision (ICD-10) [22]; mild (IQ 50–69), moderate (IQ 35–49), severe (IQ 20–34) and profound (IQ < 20).

Visual impairment was defined as an acuity of not more than 0.3 in the best eye with correction, and severe visual impairment was defined as an acuity of not more than 0.1 in the best eye with correction or the presence of functional blindness. Hearing impairment included sensorineural impairment or deafness, unilateral or bilateral. Epilepsy was defined as epilepsy under treatment according to the medical records. Speech was classified with the Viking Speech Scale (VSS) [23]; level I not affected speech, II imprecise speech, III unclear speech and IV no understandable speech.

Neuropsychiatric disorders in this study included ASD and ADHD. These diagnoses were derived from the medical records and had been coded according to ICD-10. Three autism spectrum diagnoses were found: autistic disorder, atypical autism and Asperger syndrome. ASD is used here for all three categories.

### Procedure

**Fig. 1:**
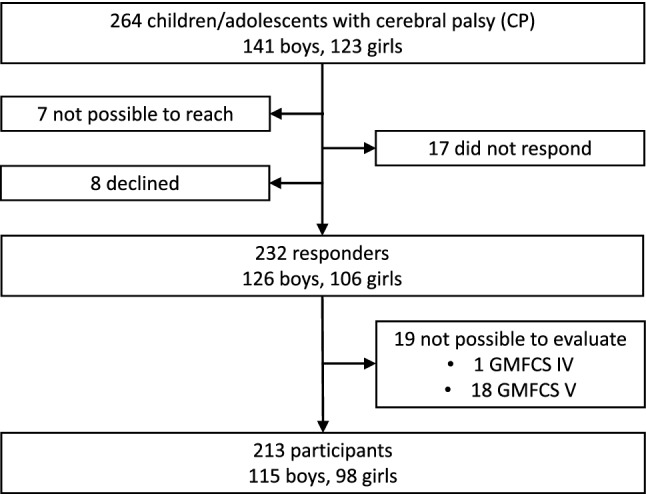
Flowchart of the study participants. The population-based group consisted of 264 children with cerebral palsy (CP) from the CP register of western Sweden. Screening questionnaires were obtained for 232 children, but 19 questionnaires were not possible to evaluate leaving the 213 study participants. (GMFCS Gross Motor Function Classification System)

Between January 2013 and December 2016 all parents of the 264 children with CP were invited to participate by completing a comprehensive combined questionnaire. The parents of 101 children were asked at a visit to the Regional Rehabilitation Centre, 156 were contacted through telephone, while the parents of seven children were not possible to reach in person or by phone. The latter received only a written invitation. The parents of eight children declined to participate. Thus, 256 questionnaires were sent out. The parents of 232 children responded either directly (*n* = 169) or after one or two reminders (*n* = 63). Twenty-four questionnaires were not returned despite reminders (Fig. 1).

The 232 received questionnaires were completed by either the mother (*n* = 131), the father (*n* = 31), both parents (*n* = 69), or the foster mother (*n* = 1). The median age at screening was 12 years 11 months (range 8 years 4 months—17 years 10 months).

### Assessments

The combined questionnaire consisted of validated screening tools used in the Bergen Child Study [24]—SDQ (Strengths and Difficulties Questionnaire), ASSQ (Autism Spectrum Screening Questionnaire), SNAP-IV (Swanson, Nolan and Pelham) and 21 extra items—with addition of two instruments covering questions pertaining to children with ID: DBC (Developmental Behaviour Checklist) and ABC (Autism Behavior Checklist), altogether 282 items (Table 1). All items were possible to score on a 3-point scale (0 = not true, 1 = somewhat true and 2 = certainly true), except for ABC where items were judged not true/true. As a principle, the lowest established cut-off levels have been used to ensure high sensitivity and to compensate for single items, most commonly pertaining to motor function or speech, not possible to evaluate for some parents of some children.

**Table 1:** Screening instruments of the questionnaire

Instrument	Items	Cut-off	Scores
SDQ (Strengths and Difficulties Questionnaire)	25		Not true 0/somewhat true 1/certainly true 2
including SDQ subscale hyperactivity-inattention^b^	(5)	6	Not true 0/somewhat true 1/certainly true 2
ASSQ (Autism Spectrum Screening Questionnaire)^a^	27	15	Not true 0/somewhat true 1/certainly true 2
Plus ASSQ-REV extended version	18		Not true 0/somewhat true 1/certainly true 2
SNAP-IV (Swanson, Nolan and Pelham)	30		Not true 0/somewhat true 1/certainly true 2
with subscales inattention and hyperactivity/impulsivity^b^	(9/9)	6/9 items scored as 1 or 2 in the two subscales, respectively	
DBC (Developmental Behaviour Checklist)	96		Not true 0/somewhat true 1/certainly true 2
including DBC-ASA (Autism Screening Algorithm)^a^	(29)	17	
including DBC-HI (Hyperactivity Index)^b^	(6)	7	
Extra items	21		
ABC (Autism Behavior Checklist)^a^	57	45	Not true/true, scoring according to algorithm (Krug et al. 1980)
SDQ function	8		
Open questions	(2)		
Total items	282		

The instruments used in the screening for autism spectrum disorder (ASD) and attention-deficit/hyperactivity disorder (ADHD), respectively, are marked

^a^Instruments assessing ASD

^b^Instruments assessing ADHD

The SDQ is a brief emotional and behavioural screening questionnaire for children and adolescents [25]. The version for parents of 4–17 year olds was used. The hyperactivity/inattention subscale consisting of 5 items was used with 6 as a cut-off level for screen positivity for ADHD [26].

The ASSQ is a widely used autism spectrum screening instrument and consists of 27 items [27]. Also, the 18 items in the extended version (ASSQ-REV) [28] were included in the questionnaires, but not reported in this paper due to lack of a validated cut-off level. For ASSQ a cut-off level of 15 (of a possible maximum of 54) was used [29].

The SNAP-IV includes the diagnostic symptoms for ADHD and oppositional defiant disorder (ODD) [30]. We defined the cut-off as 6/9 items scored as “somewhat true” or “certainly true” in the two sub-scales of inattention and hyperactivity/impulsivity, respectively [26].

The DBC is a suite of instruments for the assessment of behavioural and emotional problems in developmental and intellectual disabilities [31]. The DBC Autism Screening Algorithm (DBC-ASA) is a 29-item subscale with a cut-off level of 17 [32]. The DBC Hyperactivity Index (DBC-HI) is a 6-item subscale for hyperactivity described in a pilot study [33], and we decided to use 7 as a cut-off level for ADHD.

The ABC was developed to measure levels of autistic behaviour in individuals with severe disabilities [34]. The 57 items were weighted as originally between 1 and 4 points, and a total score of 45 was used as cut-off level [9].

In summary three scales were used to define ASD screen positivity (ASSQ, DBC-ASA and ABC) and three scales were used to define screen positivity for ADHD (SDQ hyperactivity/impulsivity, SNAP-IV and DBC-HI) (Table 1). Since the scales are targeting children at different intellectual levels, a child was considered screen-positive if at least one out of three scales for ASD and ADHD, respectively, reached cut-off levels. A child was considered screen-negative if all three scales for ASD and ADHD, respectively, were below cut-off.

Not all items had been completed in all questionnaires. Overall, data were missing in nearly 9% of all items. The instruments used differed in this respect; in SNAP 11% of the items were not completed, in ASSQ 11%, in SDQ 9%, in DBC 8% and in ABC 7%. For most children the questionnaire was applicable. In four out of five questionnaires a maximum of ten items out of all 282 were uncompleted. If less than three quarters of all items were completed in all three scales for ASD and ADHD, respectively, and no scale reached cut-off level, the questionnaire was considered not possible to evaluate.

### Ethics

The study was approved by The Regional Ethical Review Board in Gothenburg, ref 398–12.

### Statistical analysis

The aims of this study were descriptive, and statistics were used to compare groups. For the association between categorical variables, the *χ*^2^ test for independence was used, and for the comparison in a group with an ordinal scale, the Cochran–Armitage *χ*^2^ test for trends was used. A *p* value of < 0.05 was regarded as statistically significant.

The population-based basis is described in Table 2, where the original population is compared with the responders and the screening participants. To assess differences between responders and non-responders the *χ*^2^ test for independence was used. In Table 3 the results from the screening are compared with already identified diagnoses in the screening participants, for ASD and ADHD, respectively, and by different background factors. Main findings from Table 3 are also presented visually in Fig. 3.

**Table 2:** The distribution of sex, gestational age, cerebral palsy (CP) type, gross motor function and associated impairments of the total population group of children with CP, of the responders, and of the study participants

	Population	Responders	Screening participants
	*n* = 264	*n* = 232	*n* = 213
	*n* (%)	*n* (%)	*n* (%)
*Sex*			
Male	141 (53)	126 (54)	115 (54)
Female	123 (47)	106 (46)	98 (46)
*Gestational age*			
Week 23–27	26 (10)	21 (9)	20 (10)
Week 28–31	30 (11)	23 (10)^a^	22 (10)
Week 32–36	45 (17)	40 (17)	37 (17)
Week 37–42	163 (62)	148 (64)	134 (63)
*CP type*			
Unilateral spastic CP	103 (39)	89 (38)	89 (42)
Bilateral spastic CP	98 (37)	83 (36)	76 (36)
Dyskinetic CP	45 (17)	44 (19)^b^	32 (15)
Ataxic CP	18 (7)	16 (7)	16 (7)
*GMFCS*			
I	127 (48)	110 (48)	110 (52)
II	40 (15)	33 (14)	33 (15)
III	20 (8)	19 (8)	19 (9)
IV	35 (13)	31 (13)	30 (14)
V	42 (16)	39 (17)	21 (10)
*Visual impairment (VI)*			
No VI	213 (81)	189 (82)	183 (86)
VI not severe	18 (7)	15 (6)	15 (7)
Severe VI	33 (12)	28 (12)	15 (7)
*Hearing impairment*			
No	243 (92)	214 (92)	198 (93)
Sensorineural	21 (8)	18 (8)	15 (7)
*Intellectual level*			
Normal	98 (37)	90 (39)	90 (42)
Borderline	26 (10)	19 (8)^c^	19 (9)
Mild ID	57 (22)	47 (20)	47 (22)
Moderate ID	19 (7)	19 (8)	19 (9)
Severe ID	32 (12)	29 (13)	26 (12)
Profound ID	32 (12)	28 (12)	12 (6)
*Epilepsy*			
No	137 (52)	118 (51)	117 (55)
Previous	18 (7)	15 (6)	15 (7)
Active	109 (41)	99 (43)	81 (38)
*Viking Speech Scale (VSS)*			
I	122 (46)	104 (45)	104 (49)
II	58 (22)	51 (22)	51 (24)
III	17 (7)	17 (7)	17 (8)
IV	67 (25)	60 (26)	41 (19)
ASD diagnoses	47 (18)	42 (18)	42 (20)
ADHD diagnoses	55 (21)	49 (21)	49 (23)
Both ASD and ADHD diagnoses	18 (7)	18 (8)	18 (8)
No diagnosed-associated impairment	65 (25)	57 (25)	57 (27)

The three subgroups with significant differences between responders and non-responders are marked. (GMFCS Gross Motor Function Classification System; ID Intellectual Disability; ASD Autism Spectrum Disorder; ADHD Attention-Deficit/Hyperactivity Disorder)

^a^Significantly higher proportion in the non-responder group of 32 children (*p* = 0.046)

^b^Significantly lower proportion in the non-responder group of 32 children (*p* = 0.025)

^c^Significantly higher proportion in the non-responder group of 32 children (*p* = 0.015)

**Table 3:** Results of screening for autism spectrum disorder (ASD) and attention-deficit/hyperactivity disorder (ADHD) and identified diagnoses of ASD and ADHD in the same 213 children in a population-based group with cerebral palsy (CP)

		Screening	Diagnosis	Screening	Diagnosis	Screening
		positive ASD	ASD	positive ADHD	ADHD	negative ASD and ADHD
		*n* (%)	*n* (%)	*n* (%)	*n* (%)	*n* (%)
Total	213	74 (35)	42 (20)	106 (50)	49 (23)	94 (44)
*Sex*						
Male	115	45 (39)	27 (23)	62 (54)	25 (22)	46 (40)
Female	98	29 (30)	15 (15)	44 (45)	24 (24)	48 (49)
*Gestational age*						
Week 23–27	20	10 (50)	9 (45)	13 (65)	6 (30)	7 (35)
Week 28–31	22	9 (41)	4 (18)	7 (32)	4 (18)	12 (55)
Week 32–36	37	10 (27)	5 (14)	10 (27)	7 (19)	21 (57)
Week 37–42	134	45 (34)	24 (18)	76 (57)	32 (24)	54 (40)
*CP type*						
Unilateral spastic CP	89	23 (26)	15 (17)	45 (51)	24 (27)	41 (46)
Bilateral spastic CP	76	29 (38)	19 (25)	35 (46)	11 (14)	36 (47)
Dyskinetic CP	32	13 (41)	3 (9)	14 (44)	7 (22)	14 (44)
Ataxic CP	16	9 (56)	5 (31)	12 (75)	7 (44)	3 (19)
*GMFCS*						
I	110	26 (24)	15 (14)	51 (46)	27 (25)	54 (49)
II	33	15 (45)	13 (39)	19 (58)	11 (33)	14 (42)
III	19	8 (42)	5 (26)	11 (58)	4 (21)	7 (37)
IV	30	16 (53)	7 (23)	17 (57)	5 (17)	10 (33)
V	21	9 (43)	2 (10)	8 (38)	2 (10)	9 (43)
*Visual impairment (VI)*						
No VI	183	59 (32)	32 (17)	95 (52)	48 (26)	79 (43)
VI not severe	15	8 (53)	5 (33)	4 (27)	1 (7)	7 (47)
Severe VI	15	7 (47)	5 (33)	7 (47)	0 (0)	8 (53)
*Hearing impairment*						
No	198	67 (34)	40 (20)	95 (48)	45 (23)	90 (45)
Sensorineural	15	7 (47)	2 (13)	11 (73)	4 (27)	4 (27)
*Intellectual level*						
Normal	90	10 (11)	6 (7)	30 (33)	14 (16)	56 (62)
Borderline	19	7 (37)	5 (26)	9 (47)	8 (42)	10 (53)
Mild ID	47	24 (51)	12 (26)	32 (68)	18 (38)	12 (26)
Moderate ID	19	10 (53)	7 (37)	13 (68)	6 (32)	4 (21)
Severe ID	26	18 (69)	9 (35)	19 (73)	3 (12)	6 (23)
Profound ID	12	5 (42)	3 (25)	3 (25)	0 (0)	6 (50)
*Epilepsy*						
No	117	28 (24)	12 (10)	48 (41)	26 (22)	62 (53)
Previous	15	6 (40)	4 (27)	6 (40)	2 (13)	8 (53)
Active	81	40 (49)	26 (32)	52 (64)	21 (26)	24 (30)
*Viking Speech Scale (VSS)*						
I	104	24 (23)	18 (17)	45 (43)	23 (22)	53 (51)
II	51	20 (39)	11 (22)	25 (49)	16 (31)	24 (47)
III	17	6 (35)	2 (12)	10 (59)	5 (29)	7 (41)
IV	41	24 (59)	11 (27)	26 (63)	5 (12)	10 (24)

The results are shown in relation to sex, gestational age, CP type, gross motor function and associated impairments. Percentages describes the proportion of the subgroups. (GMFCS Gross Motor Function Classification System; ID Intellectual Disability)

## Results

The screening response rate was 88% (232 questionnaires out of 264). The responders represented the whole group of children with CP regarding sex, gestational age, CP type, GMFCS level, intellectual level, visual and hearing impairment, epilepsy and speech ability. Overall there were no major differences between the responders and the non-responders. Significant differences were seen in three subgroups: gestational age 28–31 weeks, dyskinetic CP and borderline intellectual functioning (Table 2).

The questionnaires of 19 children were not possible to assess due to too few completed items. They represented the most disabled children; 12 with dyskinetic CP and seven with BSCP at the most severe GMFCS levels and ID levels. They accounted for more than two thirds of all uncompleted items in the questionnaires. In the following these 19 children are excluded, leaving 213 children.

The proportion of diagnosed ASD and ADHD in the screening group of 213 children did not differ from the original group (Table 2).

### Positive screening

**Fig. 2:**
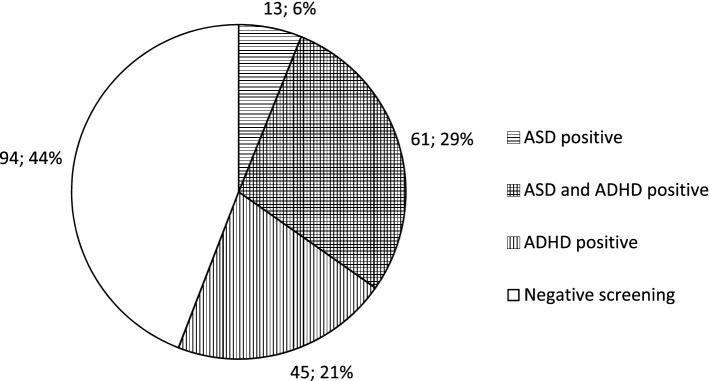
Screening outcome in a population-based group of children with cerebral palsy (CP). Results from the 213 analysed questionnaires showed one third, 74/213, screened positive for autism spectrum disorder (ASD) and half, 106/213, screened positive for attention-deficit/hyperactivity disorder (ADHD). There was a considerable overlap of 61/213, resulting in 119/213 screened positive for ASD or ADHD or both

The remaining 213 questionnaires were analysed concerning signs of ASD and ADHD. Positive screening was found in 119 children (56%) for either ASD or ADHD or both. Seventy-four (35%) screened positive for ASD and 106 (50%) for ADHD. There was an overlap of screening positive for both ASD and ADHD in 61 children (29%) (Fig. 2).

Of the 74 children who screened positive for ASD, 68 children were positive on the ASSQ, 34 on the DBC-ASA, and 26 on the ABC. Nineteen children were positive on all three screening instruments, 16 on two instruments, and 39 on only one instrument. An ASD diagnosis had been registered in 42 of the 213 children.

Of the 106 children who screened positive for ADHD, the SNAP-IV was positive for 91 children, the SDQ hyperactivity/impulsivity for 58 and the DBC-HI for 28 children. Twenty-one children were positive on all three screening instruments for ADHD, 29 on two instruments, and 56 children on only one instrument (either of the three). An ADHD diagnosis had been registered in 49 of the 213 children.

The screening results of ASD and ADHD and registered diagnoses of ASD and ADHD are shown in Table 3. Almost twice as many screened positive for ASD than were previously diagnosed, and more than twice as many screened positive for ADHD than were already diagnosed.

Of the 42 children with an ASD diagnosis, 33 screened positive for ASD (sensitivity 79%). Of the remaining nine children, five screened positive for ADHD only. Of the 49 children with an ADHD diagnosis, 42 screened positive for ADHD (sensitivity 86%). The children with ASD or ADHD diagnoses who screened negative did not differ from the children who screened positive regarding sex, gestational age, CP type, GMFCS level or other associated impairments.

### Associated characteristics

For ASD, 45 of the 115 boys (39%) and 29 of the 98 girls (30%) screened positive, and for ADHD, 62 of the 115 boys (54%) and 44 of the 98 girls (45%) (Table 3). These differences in sex distribution were not significant. Children born extremely preterm were more likely to have been diagnosed with ASD than children born from 28 gestational weeks (*χ*^2^ = 8.91; *p* = 0.003), but screening for ASD showed no significant differences between gestational age groups (Table 3).

**Fig. 3:**
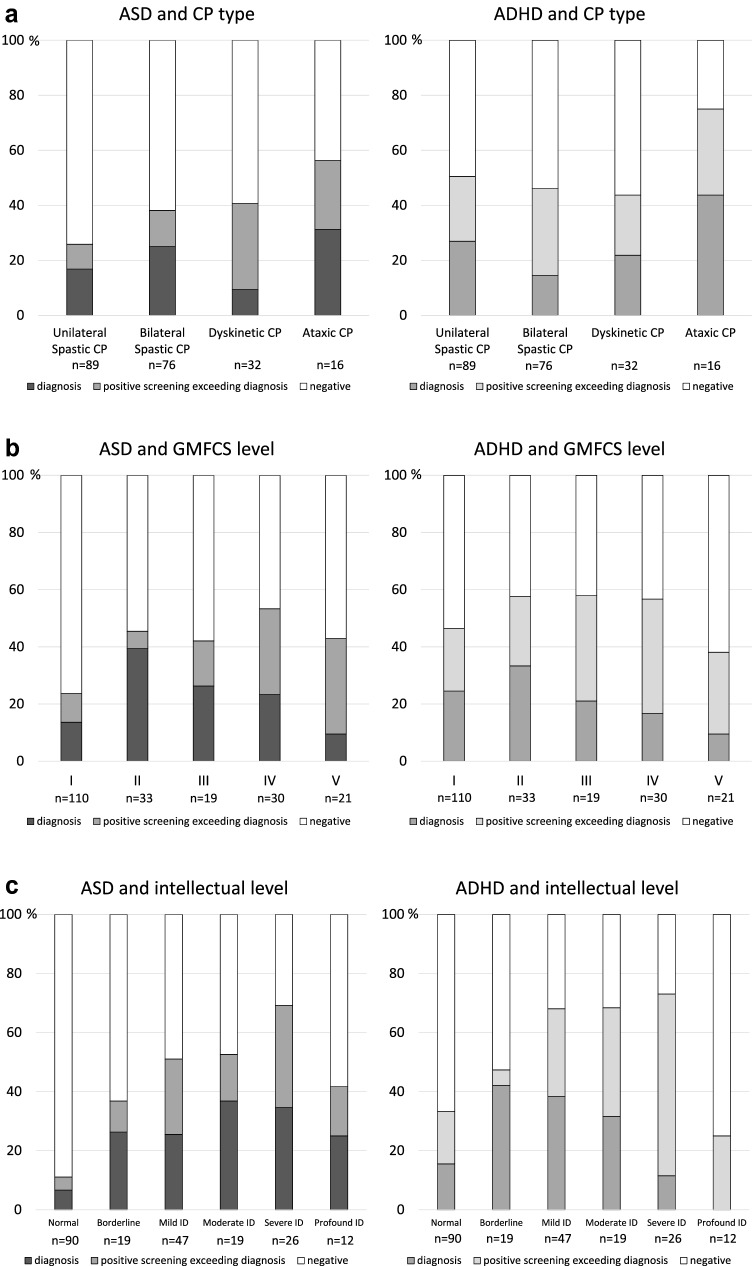
Results of screening for autism spectrum disorder (ASD) and attention-deficit/hyperactivity disorder (ADHD) in relation to identified diagnoses of ASD and ADHD in the same 213 children in a population-based group of children with cerebral palsy (CP). The darker parts of the staples represent the proportion diagnosed ASD and ADHD, respectively, and the lighter parts represent the proportion screened positive above the proportion with diagnoses, and are shown for **a** CP type, **b** gross motor function (GMFCS level) and **c** intellectual level. (GMFCS Gross Motor Function Classification System; ID Intellectual Disability). **a** Positive screening and identified diagnoses of ASD and ADHD in relation to CP type. **b** Positive screening and identified diagnoses of ASD and ADHD in relation to gross motor function (GMFCS level). **c** Positive screening and identified diagnoses of ASD and ADHD in relation to intellectual level

Positive screening for ASD and ADHD was common in all CP types (Fig. 3a, Table 3). There was an increasing occurrence of ASD screening positivity with more severe GMFCS levels (*χ*^2^_trend_ = 9.91; *p* = 0.003), while no association was seen between ADHD screening positivity and GMFCS levels (Fig. 3b, Table 3). In contrast, identified diagnoses of both ASD and ADHD decreased from GMFCS level II to V.

Excluding children with profound ID, children with more severe ID screened positive more often both for ASD (*χ*^2^_trend_ = 41.79; *p* < 0.001) and ADHD (*χ*^2^_trend_ = 21.56; *p* < 0.001) (Fig. 3c, Table 3). This association was similar in already identified ASD diagnoses. In contrast, identified ADHD diagnoses were more prevalent towards less severe ID.

Children with epilepsy screened positive more often than children without epilepsy, for ASD (*χ*^2^ = 12.36; *p* < 0.001) as well as ADHD (*χ*^2^ = 10.89; *p* = 0.001) (Table 3). Children with less speech ability more often screened positive for ASD (*χ*^2^_trend_ = 15.59; *p* < 0.001) as well as ADHD (*χ*^2^_trend_ = 5.33; *p* = 0.021), although no associations between diagnosed ASD and/or ADHD and speech ability were seen (Table 3).

## Discussion

In this population-based screening study, we found that a very large proportion of children with CP screened positive for ASD or ADHD or both. One third of the children screened positive for ASD and half of the children for ADHD. The rates were higher than expected; about double of already identified diagnoses in the same group of children.

This screening study was designed to have high sensitivity, but with stricter cut-off levels the proportion screening positive would still have been high. For example, if an ASSQ cut-off of 17 rather than 15 had been used, the ASD screen positivity rate would still be 32%, instead of 35%. Nevertheless, the questionnaire did not identify all children diagnosed with ASD and/or ADHD. For ADHD, three of the nine children with a registered diagnosis who screened negative were on treatment with stimulants, which may have led to a considerable decrease in ADHD symptoms.

The DBC-HI has been evaluated in a pilot study resulting in suggestions for two different cut-off levels used for different purposes [33], and we decided to use 7 which was said to represent the clinically significant range. Had we used the lower suggested cut-off of 5, the ADHD screen positivity rate would have been 52%, instead of 50%. Gargaro et al. also emphasise that children with ASD only often show high, but sub-clinical, levels of hyperactivity symptoms [33]. In our study we found a considerable overlap between ASD and ADHD screening positivity of 29%, and only 6% screened positive for ASD only, i.e. the majority of children screened positive for ASD also screened positive for ADHD.

One important finding was that the instruments used were not appropriate for the most severely disabled children, i.e. children at GMFCS level V and profound ID. In 19 children the questionnaires were impossible to evaluate, due to too few completed items. Children with very severe impairments may not have enough abilities or expressions for the questionnaires to be feasible. In the remaining questionnaires regarding the children at GMFCS level V, fewer screened positive than in the less disabled children. In the study by Bjørgaas et al. [14], the same problem was encountered, therefore all with GMFCS level V were excluded. There is a need for finding other solutions to evaluate this group.

ASD as well as ADHD screen positivity increased the more severe other impairments; ID, impaired speech ability and epilepsy. ASD screen positivity also increased with more severe motor impairment, while this association was not seen for ADHD. The comparison with already identified diagnoses of ASD and ADHD revealed a clear difference since diagnoses most often decreased with increasing other impairments. The gap between screening positivity and identified diagnoses was bigger the more severe other impairments, which may reflect diagnostic difficulties in children with several impairments.

There was no significant male preponderance for either ASD or ADHD in this study, in contrast to the screening findings of the Bergen Child Study [26, 29].

### Strengths and limitations

Participants in this study all belonged to a well-defined population-based group of children with CP, from the CP register of western Sweden [1, 2]. The response rate was high, 88% of all children, representative regarding sex distribution, CP types, GMFCS levels and associated impairments. This argues in favour of the results being generalisable. Another strength of the study is that we used three complementary instruments to screen for ASD and ADHD, respectively, and also included instruments developed for children with ID. This broader approach has most likely increased the possibility to capture signs of ASD and ADHD.

A limitation of the study is that information on the children came only from one source, the parents. This source of information is probably sufficient for most children with CP, but it may be hard for some parents to accept their children’s impairments, seeking other explanations for their problems. Conversely, some parents may look for impairments that more “objective” assessment would not pick up on. Additional teacher ratings would have been valuable. Another limitation is that the DBC, apart from the other screening instruments, has not been validated in Swedish, although that has been done in nearby countries.

Another important point is that all children who screened positive would likely not meet full diagnostic criteria for ASD and/or ADHD after comprehensive clinical assessment. Further investigations are needed in these children, but even in children where diagnostic criteria are not met, the symptoms could still be impairing and important to consider when planning support.

### Implications

Our findings support the hypothesis that the prevalence of neuropsychiatric impairments in children with CP is underestimated. Children with one neurodevelopmental disability are at greater risk of also having other neurodevelopmental disabilities [35] and should therefore be assessed for a range of possible problems (ESSENCE, Early Symptomatic Syndromes Eliciting Neurodevelopmental Clinical Examinations) routinely. Early diagnosis and support have been proven to give a better prognosis for children with ASD [36], and children with ADHD also benefit from early identification and treatment [37]. A child with combined disabilities will benefit from more adequate and effective treatment, hopefully leading to a better level of functioning and participation. Parents may also benefit from early support with less stress and more knowledge about how to take care of their child. From a community perspective, it is important to recognise the common combination of CP and neuropsychiatric impairments to provide better services [17].

## Conclusion

This population-based study of children with CP indicates a higher prevalence of ASD and ADHD than previously described; one third of the children with CP may have ASD and half may have ADHD. These findings require further follow-up investigations pertaining to identifying the most suitable instruments for neuropsychiatric evaluation in children with CP, particularly for the more severely disabled children. It would also be valuable to assess the screen positive children without diagnoses more in depth to come closer to the true prevalence of ASD and ADHD in children with CP.
